# Recent Advances in Manufacturing Innovative Stents

**DOI:** 10.3390/pharmaceutics12040349

**Published:** 2020-04-13

**Authors:** Natalia Beshchasna, Muhammad Saqib, Honorata Kraskiewicz, Łukasz Wasyluk, Oleg Kuzmin, Oana Cristina Duta, Denisa Ficai, Zeno Ghizdavet, Alexandru Marin, Anton Ficai, Zhilei Sun, Vladimir F. Pichugin, Joerg Opitz, Ecaterina Andronescu

**Affiliations:** 1 Fraunhofer Institute for Ceramic Technologies and Systems IKTS, Maria-Reiche-Str. 2, 01109 Dresden, Germany; muhammad.saqib@ikts-extern.fraunhofer.de (M.S.); joerg.opitz@ikts.fraunhofer.de (J.O.); 2 Balton Sp. z o.o. Modlińska 294, 03-152 Warsaw, Poland; honorata.k@wp.pl (H.K.); lukasz@balton.pl (Ł.W.); 3 VIP Technologies, Prospect Academicheskiy 8/2, 634055 Tomsk, Russia; ostk1961@mail.ru; 4 Department of Science and Engineering of Oxide Materials, Faculty of Applied Chemistry and Materials Science, University Politehnica of Bucharest, Spl. Independentei 313, 060042 Bucharest, Romania; oana_cristina_duta@yahoo.com (O.C.D.); denisaficai@yahoo.ro (D.F.); zghizdavet@gmail.com (Z.G.); ecaterina.andronescu@upb.ro (E.A.); 5 Department of Hydraulics, Hydraulic Machinery and Environmental Engineering, Faculty of Power Engineering, University Politehnica of Bucharest, Spl. Independentei 313, 060042 Bucharest, Romania; alexandru.marin@upb.ro; 6 Academy of Romanian Scientists, Spl. Independentei 54, 050094 Bucharest, Romania; 7 Research School of High-Energy Physics, Tomsk Polytechnic University, Lenin Avenue 30, 634050 Tomsk, Russia; Zhilei.SUN@mail.ru

**Keywords:** stent, stent coating, titanium oxynitride coating, drug-eluting stent, bioresorbable stent stent manufacturing

## Abstract

Cardiovascular diseases are the most distributed cause of death worldwide. Stenting of arteries as a percutaneous transluminal angioplasty procedure became a promising minimally invasive therapy based on re-opening narrowed arteries by stent insertion. In order to improve and optimize this method, many research groups are focusing on designing new or improving existent stents. Since the beginning of the stent development in 1986, starting with bare-metal stents (BMS), these devices have been continuously enhanced by applying new materials, developing stent coatings based on inorganic and organic compounds including drugs, nanoparticles or biological components such as genes and cells, as well as adapting stent designs with different fabrication technologies. Drug eluting stents (DES) have been developed to overcome the main shortcomings of BMS or coated stents. Coatings are mainly applied to control biocompatibility, degradation rate, protein adsorption, and allow adequate endothelialization in order to ensure better clinical outcome of BMS, reducing restenosis and thrombosis. As coating materials (i) organic polymers: polyurethanes, poly(ε-caprolactone), styrene-b-isobutylene-b-styrene, polyhydroxybutyrates, poly(lactide-co-glycolide), and phosphoryl choline; (ii) biological components: vascular endothelial growth factor (VEGF) and anti-CD34 antibody and (iii) inorganic coatings: noble metals, wide class of oxides, nitrides, silicide and carbide, hydroxyapatite, diamond-like carbon, and others are used. DES were developed to reduce the tissue hyperplasia and in-stent restenosis utilizing antiproliferative substances like paclitaxel, limus (siro-, zotaro-, evero-, bio-, amphi-, tacro-limus), ABT-578, tyrphostin AGL-2043, genes, etc. The innovative solutions aim at overcoming the main limitations of the stent technology, such as in-stent restenosis and stent thrombosis, while maintaining the prime requirements on biocompatibility, biodegradability, and mechanical behavior. This paper provides an overview of the existing stent types, their functionality, materials, and manufacturing conditions demonstrating the still huge potential for the development of promising stent solutions.

## 1. Introduction

Stenting of arteries became a common treatment of cardiovascular medicine, enabling the re-opening of the narrowed vessels and restoring the normal blood flow. Current technologies, especially very promising and rapid development of drug eluting stents (DES), demonstrate good efficacy with a low rate of treatment failure, making it possible to also expand the stent application to patients with complicated diseases [1]. However, complications, such as in-stent restenosis, late thrombosis, local chronic inflammation, and re-occlusion rates, are still results of stent implantations [2], so that further development of stent devices and deep analysis of their long-term stability and failure mechanisms is necessary. The restenosis rate of high-risk patients having small vessels, diabetes and long diffusion diseased arteries still remains unacceptably high ((30–60%) in bare-metal stents (BMS) and (6–18%) in DES) [1], demonstrating the challenge of stent technology and the need for new, more safe solutions for all patient categories.

The first generation of stents, BMS, usually fabricated from stainless steel (316L), cobalt–chromium (Co–Cr) and platinum–iridium (Pt–Ir) alloys, tantalum (Ta), or nitinol (Ni–Ti) have shown numerous problems leading to tissue hyperplasia, in-stent restenosis and the necessity to explant them or to keep them as a foreign body during the whole life. These considerations pushed the development of coated stents, DES, and biodegradable stents (BDS) [3]. Providing an overview of the stent technology in the treatment of ischemic stroke, describing the commonly used stents, and defining the development trends in their fabrication technologies, Yoon et al. [4] have shown the potential of coated stents, DES [5,6], and BDS for future applications. These kinds of stents will be considered in-depth in this review article. An ideal stent should possess properties as formulated by Mani et al. [7]: (1) ability to be crimped on the balloon catheter; (2) good expandability ratio; (3) sufficient radial hoop strength and negligible recoil; (4) sufficient flexibility; (5) adequate radiopacity/magnetic resonance imaging (MRI) compatibility; (6) high thromboresistivity; (7) absence of restenosis after implantation; (8) non-toxicity; and (9) drug delivery capacity. The optimization of mechanical, physical–chemical, and biological stent properties is challenging and should lead to the achievement of the above-mentioned characteristics. Especially critical are the biological aspects related to the adhesion of salts, proteins, cells, and microorganisms on the stent surface causing undesired effects like encrustation, biofilm formation, inflammation, and stent failure. In the forthcoming sections, the authors discuss the correlation of such events with the stent surface properties, highlighting the benefits of using different kinds of stents.

## 2. Coated Stents

### 2.1. Coating Types and Materials

**Figure 1:**
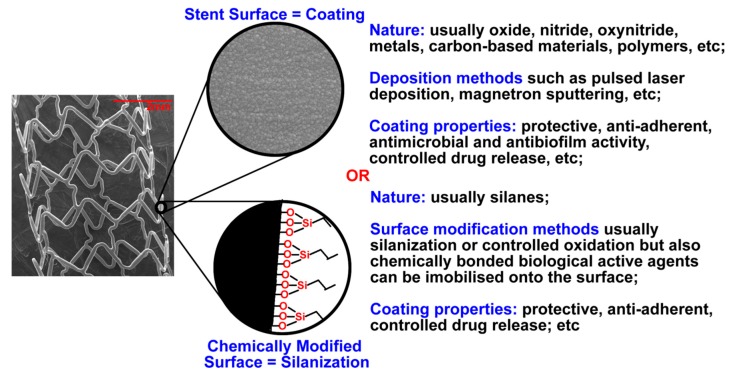
Stents surface modification techniques.

Several classes of materials have been tested as potential coatings for stent manufacturing (Figure 1). The stent surface can be modified by using oxides and nitrides of metals, whereby metals and polymers are deposited using different physical–chemical methods, such as magnetron sputtering, pulsed laser deposition, matrix-assisted pulsed laser evaporation, etc. Chemical surface modification can also be assured by molecular layer deposition, for instance, promising silanization technology utilizing commercially available silanes (ethyltrietoxysilane, octyltriethoxysilane, vinyltrimethoxysilane, n-octadecyltriethoxysilane, phenyltriethoxysilane, (3-aminopropyl) triethoxysilane, (3-mercaptopropyl)triethoxysilane, etc.), rich on diverse functional groups helping to achieve the desired stent properties. Considering the advances in the field of drug delivery, coatings can modulate the delivery rate of the biological active agents loaded into/onto them [8,9]. For example, in the patent US9101689B2 [10], a stent with created reservoir regions containing an active ingredient, e.g., actinomycin D or taxol, demonstrates the possibility to release the active ingredients in different rates. The tissue- and blood-biomaterial interfaces are particularly important for optimizing any cardiovascular stent. The inner stent surface interacts with flowing blood while the outer surface makes contact with vessel tissue. Stent materials should be biocompatible and stable to allow the adherence of monolayer of specific cells while preventing the adhesion of minerals, proteins, and multi-layered cells at the same time. The surface can be modified in order to avoid or to reduce undesired corrosion able to disturb a stent integrity and its function [7] as well as to cause ion release, which leads to significant impact on the surrounding vascular cells.

In [11], an innovative approach of electrical surface functionalization based on engineering the electrical charge on the stent surface has been reported. The influence of electrostatic factor on the cell attachment is demonstrated as important tool for manipulation of cell response directly associated with restenosis and thrombosis. The electrical functionalization of stent material surfaces can be achieved by ionizing (high and low energy electrons, gamma), and nonionizing (ultraviolet) radiation, and estimated using pre-threshold photoelectron spectroscopy (contactless technique) and Kelvin probe atomic force spectroscopy (weakly contacting technique). Obtaining the optimal electrical properties of the stent surface can help to control their ability to attach cells and biomolecules and achieve a significant improvement of biological stent properties.

In order to achieve better properties of titanium stent surface, Nanci et al. [12] modified it by chemical etching with sulfuric acid and hydrogen peroxide, followed by silanization and, finally, protein adsorption and linking via glutaraldehyde. Hiob et al. [13], starting from the premises that all current metallic vascular prostheses highlight suboptimal biocompatibility, proposed to improve the re-endothelialization process by covalent attachment of the tropoelastin onto the plasma-activated surfaces of metallic stents. Based on the achieved results, the N-terminal tropoelastin led to the improvement of the cell attachment and proliferation while the C-terminal tropoelastin-based constructs resulted in the diminishing of their activity. The goal of the study realized by Ravenscroft-Chang et al. [14] was to investigate the morphological and physiological effects of surfaces modified by self-assembled monolayers of fluorinated (hydrophobic) and amine-containing (hydrophilic) silanes as models for implant coatings. The authors focused on behavior of intracellular Ca^2+^ ions in relation to their important role in regulating heart cell function.

When discussing metals, the controlled surface oxidation is important because the intermediary oxide/hydroxide layer allows a better, more durable deposition layer onto these surfaces. Some of the most used activation procedures involved chemical surface modifications, which can even be induced by physical treatments, such as plasma treatment [15,16,17]. Coated stents have been developed to improve the properties of BMS providing better biocompatibility, non-toxicity, suitable surface roughness, and surface free energy, regulating the ability of a stent surface to adsorb biological molecules and cells, ensuring chemical stability by regulating the corrosion rate [4] or providing desirable biodegradable properties and serving as platforms for drug delivery. Various materials have been evaluated for applications as stent coatings, as discussed in several publications [4,17]. For the enhancement of biocompatibility, coating materials of better compatibility as compared to the material of stent struts have been used. For example, inorganic coating materials, such as titanium nitride, titanium oxide, or titanium oxynitride have been applied for stainless steel stents. A large variety of functional coatings for drug delivery based on biodegradable polymers, micro- and nanostructured metal, and ceramic layers have been developed. These modifications can significantly increase the success of stenting. There are three types of materials for coating fabrication: inorganic compounds, polymers, and endothelial cells. Inorganic materials and polymers can be used for creation of porous coatings.

#### 2.1.1. Organic Coatings

Polymer materials are used as stent coatings both with and without drug elution, with different success [18,19,20,21]. The main problem of biodegradable and non-biodegradable polymer layers lies in their degradation products, arising as result of contact with biological fluids and ability to trigger inflammation followed by thrombosis formation. Susceptibility of polymers to fractures can lead to the release of materials fragments into the blood flow, provoking a danger to close some narrowed arears of the damaged vessels.

Poly(ethylene) (PE), polyurethanes (PUR), poly(glycolide) (PGA) and polylactides (PLA) have been evaluated as stent coating materials [22], being already used for implants or other medical devices [23,24,25,26,27,28,29,30,31,32,33,34]. While polyurethanes are well established as scaffold materials for vascular grafts due to their excellent hemocompatibility [23,24,25,26], PGA is commonly used as suture material for different surgical applications [27]. Furthermore, PGA-containing scaffolds blended with poly(-caprolactone) (PCL) [28] are used for PGA-based drug delivery systems [29,30,31]. PLA has been intensely tested as temporary stent material in cardiology due to its long track records of in vivo biocompatibility [32,33,34]. Bognar et al. [19] evaluated three types of polyurethanes (carbothane, tecothane, and chronoflex) deposited on stainless steel stent surfaces (L316) by dipping into a solution. The performed experiments have shown a suitable adherence of PUR coating to the stent surface, as well as improved biocompatibility and long-term stability comparing to the non-coated stents [18,35,36,37,38]. Since the beginning of using polyurethane as a stent coating material (early 1990s), such stents were applied in coronary artery perforation [37], for treatment of esophagorespiratory fistulas [38,39], recurrent benign urethral stricture [40], malignant biliary [41,42], esophageal [43,44] or gastroduodenal obstruction [45], and irresectable esophageal carcinoma [46].

Further examples of polymer applications as stent coatings (such as non-biodegradable: poly(methacryloyl phosphorylcholine-co-laurylmethacrylate), poly(n-butyl methacrylate), poly(ε-caprolactone), poly(ethylene terephthalate) and silicone, and biodegradable: poly(lactic acid)-PLA, poly(glycolic acid)-PGA and their co-polymer-PLGA)) can be found in [20,21,47,48,49].

A method for preparing a nitric oxide-generating adherent coating, comprising of polyphenol compounds, organic selenium or sulfur compounds and soluble copper salts is disclosed in the invention US2017246353 [50]. The nitric oxide-generating material prepared by the method possesses the capability of scavenging free radicals and catalyzing S-nitrosothiols to produce nitrogen monoxide, which is known to reduce the risks of thrombosis, inflammation, and restenosis related to the stent applications.

#### 2.1.2. Bio-Based Coatings

Special stent coatings based on biological materials may be very attractive. Primarily, these are endothelial cells placed on the stent surface before its implantation with the aim to proliferate, differentiate, release growth, and, finally, inhibit thrombosis and neointimal hyperplasia. Several attempts to seed endothelial cells on medical grafts have been made, but all of them were unsuccessful so far [7,51,52]. The effect of stents coated with antibodies to endoglin (ENDs) on coronary neointima formation is the aim of the study [53]. The results demonstrated that endoglin antibody-coated stents reduce restenosis in the porcine model and may be considered as a new approach to prevent restenosis.

Garg and Serruys [54] are reporting about the application of CD34+ antibodies as bio-coating on stainless steel BMS (OrbusNeich, Fort Lauderdale, FL, USA). Unfortunately, this study did not demonstrate any significant success because of non-specific phenotype endothelial progenitor cells (EPCs) of CD34+ markers. Further development of novel coatings on stainless steel stents consisting of vascular endothelial growth factor (VEGF) and anti-CD34 antibody are presented in [55].

The invention CN109663151 [56] relates to a preparation method and application of an amino-rich stent material modified by copper 4-carboxyphenyl porphyrin. This technology belongs to the biological surface modification and contributes to the improvement of biocompatibility, repair of endothelial cells and inhibition of excessive proliferation of smooth muscle cells on the stent surfaces.

The invention WO2014049604 [57] provides a stent containing biofilms or a suspension of microorganisms selected from different kinds of bacteria (for example *pseudomonas aeruginosa, streptococcus, staphylococcus, salmonella, clostridia, mycobacterium bovis, Bacille Calmette Guerin - BCG*) useful in cancer therapy. The microorganisms may be attenuated in their virulence factors and with cloned genes encoding specific proteins with anticancer activity.

#### 2.1.3. Inorganic Coatings

There are a lot of inorganic materials potentially capable of improving the properties of the implant surface. Prospective inorganic materials for manufacturing of stent coatings are oxides, nitrides, silicide and carbide, noble metals, hydroxyapatite-based materials, diamond, and diamond-like carbon [58,59,60,61,62,63,64,65,66].

##### Titanium Oxide and Titanium Oxynitride Coatings

Titanium oxide-based layers are the most promising coatings for cardiovascular stent applications among all inorganic materials. The conception of drug and polymer free bioactive stent (BAS) that interferes with the healing process is related to nitrogen-doped titanium oxide (TiO_x_N_y_) coatings. The idea was firstly developed by Hexacath (Paris, France) and generalized in [67], where the results of comparative tests of BAS to paclitaxel-eluting stents (PES) in 425 patients with acute myocardial infarction were presented. The stainless steel bioactive stent Titan2 (Hexacath, Paris, France) coated by plasma enhanced vapor deposition of titanium in a mixed nitrogen-oxygen atmosphere is able to inhibit platelet aggregation, minimize fibrin deposition, reduce inflammation, and promote healing. A new generation of titanium oxynitride-coated stents TiO_x_N_y_ and TITAX-AMI have been proven to be safe and successful in reducing in-stent restenosis (ISR) in recent clinical trials [68,69,70,71] and are available at the market. The clinical outcomes of these stents were comparable to those of DES (TAXUS-Liberte Stent).

The physical-chemical and biological properties of TiO_x_N_y_ films depend on the deposition technique, the concentration of N/O [60] and on dopants incorporated into the coating [61]. For example, the implantation of phosphorus into titanium oxide film has been shown to improve the thromboresistivity of stents [61]. The data presented in [72] demonstrate that titanium oxide films doped with tantalum and formed by the plasma immersion ion implantation and deposition (PIIID) technique possess significantly better hemocompatibility comparing to undoped coatings. The TiO_x_N_y_-surfaces fabricated by a microwave-assisted process show a higher photocatalytic activity of nitrogen-containing films providing better suitability for medical applications [73]. The investigative results of titanium-oxynitride (TiO_x_N_y_) coatings deposited on L316 stainless steel by reactive magnetron sputtering are presented in [74]. The described coatings are highly biocompatible, possess a negatively charged surface and negative zeta potential, prevents in vitro adhesion of salts on the surface, and alters the surface wettability. The morphological and biological properties of the coatings can be varied by controlling the oxygen to nitrogen ratio depending on the desired surface performance. The obtained results show that plasma technologies allow for manufacturing coatings with unique structures and properties, making it possible to modify the stent surface with regard to the patient’s needs (individualized medicine).

Nitric oxide (NO) is one of the most important molecules in biological systems and plays a critical role in pathophysiology and disease. This resulted in the development of new therapeutic strategies and novel donors of nitric oxide [75,76,77]. The titanium oxynitride films, formed using a high-tech process, combines the benefits of two components: titanium oxide and nitric oxide (NO) in its atomic form. Structural features of nitrogen-containing films of titanium dioxide were studied in [78]. The results show that the films consist of anatase and rutile mixture with nanostructure (mean crystallite size from 10 to 20 nm) and nitrogen as nitric oxide (NO) located at intergranular positions in the form of an NO two-dimensional layer located at the TiO_2_ grains boundary. This suggests that TiO_x_N_y_ films can serve as the depot of nitrogen oxides directly in the field of pathology if they serve as stent coatings. In this case, it is possible to predict the following mechanism of interaction of the TiO_x_N_y_ coating with a biological system: (i) titanium and its oxides increase the corrosion resistance of implants and reduce the risk of inflammation. (ii) Titanium oxide inhibits the electron transfer from fibrinogen to the surface, reducing platelet aggregation and fibrinogen coagulation; and (iii) nitric oxide (NO) released from the coating performs the necessary biological functions, promotes endothelialization, activating the growth of endothelial cells. It is important to study the structural features and properties of nitrogen-containing films of titanium dioxide formed by ion-plasma methods and to establish the relationship between the features of the microstructure of the films and the conditions of their deposition [74,75,76,77,78]. The problem of applying a uniform, stable coating that retains high physicochemical and adhesive properties after opening the stent remains open [60]. The studies [78,79] provide an impact of nitrogen content in the reactive magnetron plasma discharge on the structure and properties of deposited TiO_x_N_y_ films. The increase of nitrogen content up to 3N_2_/O_2_ mass flow ratio leads to predominant formation of rutile phase in deposited films. The presence of nitrogen in plasma inhibits the growth of TiO_2_ anatase phase and leads to reduction of film’s grain sizes up to four times. In addition, the N_2_/O_2_ ratio influences significantly the further physical-chemical properties of TiO_x_N_y_ coated stent surface, for example their electric potential, roughness wettability, and surface energy.

##### Diamond-Like Carbon (DLC) Coatings

The properties of diamond-like carbon (DLC) surfaces and their suitability for medical applications are presented in review [80]. This material has been reported to possess the required mechanical and surface characteristics, and good biocompatibility [62,63] being successfully used as coating material for medical grafts [68]. In vitro results demonstrate that DLC and doped DLC films can prevent thrombus formation in vascular applications and show good bio- and hemocompatibility [81,82]. Characterization of cobalt-chromium stents covered by a nanostructured and homogeneous DLC film deposited by PVD inhibit fibrin deposition and platelet activation [62]. This lead to more complete and homogeneous endothelialization without triggering thrombotic clots. Antibacterial effects of DLC and doped DLC have been documented in [83]. Several reviews analyze DLC coatings and coating strategies.

In [84], calcium- and phosphorus-doped DLC films were fabricated by plasma immersion ion implantation and deposition. Doping DLC with calcium or phosphorus enhances the surface blood compatibility. Silicon (Si) as dopant in the DLC film reduces inflammatory activity as compared to the uncoated materials [85]. The increased thromboresistivity of DLC films deposited by the radio frequency plasma enhanced CVD method and treated with plasma of oxygen gas has been discussed in [86]. The experiments show the strong dependency of DLC properties on the deposition conditions and doping effects [80,87,88,89]. The results concerning in vivo testing and medical studies of DLC coated stents can be found in several papers [80,90,91,92].

Based on the reviewed literature, application of the diamond-like carbon coatings on stents have been claimed to have a positive outcome on their hemocompatibility and antithrombogenicity. In order to confirm these data, more comparative studies have to be performed. The long-term performance of carbon-based materials regarding the degradation behavior in vivo has to be studied.

##### Other Inorganic Coatings

Gold is known to be corrosion-resistant and well tolerated by the organism [93]. It was applied as a stent coating, however, the clinical outcome of the gold-coated stainless steel stents was not satisfactory [7]. As a biocompatible inert ceramic coating material for stents, iridium oxide has been studied [7,21]. Unfortunately, hydrogen peroxide produced during its corrosion was found to be harmful for the artery and caused inflammatory reactions [94,95]. Due to its anti-thrombogenic properties, amorphous silicon carbide (SiC) was applied as a stent coating [7,96] being able to reduce deposition of platelets, leukocytes, and monocytes over a stent [64,65,66]. However, the luck of experimental and especially clinical data related to this material makes further research of its properties necessary for future applications. In [97], the results of the first-in-man trial of SiO_2_-coated BMS (Axetis, Zug, Switzerland) showed insufficient suppression of neointimal hyperplasia.

Hydroxyapatite (HAp) as a stent coating material deposited by sol-gel (SG) technique was analyzed in [98]. The nanoporous designed HAp film with a thickness of 0.1–1.0 μm was used for drug encapsulation. The samples showed very good biocompatibility but no significant improvement in the histological characterization. The polymer-free deposited sirolimus layer on the top of the hydroxyapatite film as a stent coating resulted in less local toxicity and faster healing response comparing to the uncoated stainless steel stent (VESTAsyn, MIV Therapeutics, Atlanta, GA, USA). The invention CN109432493 [99] relates to a nano-hydroxyapatite-coated porous stent, a preparation method and its application. The stent includes a three-dimensional (3D)-printed porous titanium matrix covered with nano-hydroxyapatite coating and microporous network TiO_2_ layer.

## 3. Bioresorbable Stents

Bioresorbable or biodegradable stents are manufactured from a material that may be dissolved or absorbed in the body. The idea of stent bioresorbability is perceived as revolutionary (stents of third generation) according to Erne et al. [100] and attracted a strong interest of both engineers and clinicians. Multiple published reviews focus on clinical, material, and technological aspects of bioresorbable stents [5,54,101,102,103,104].

**Figure 2:**
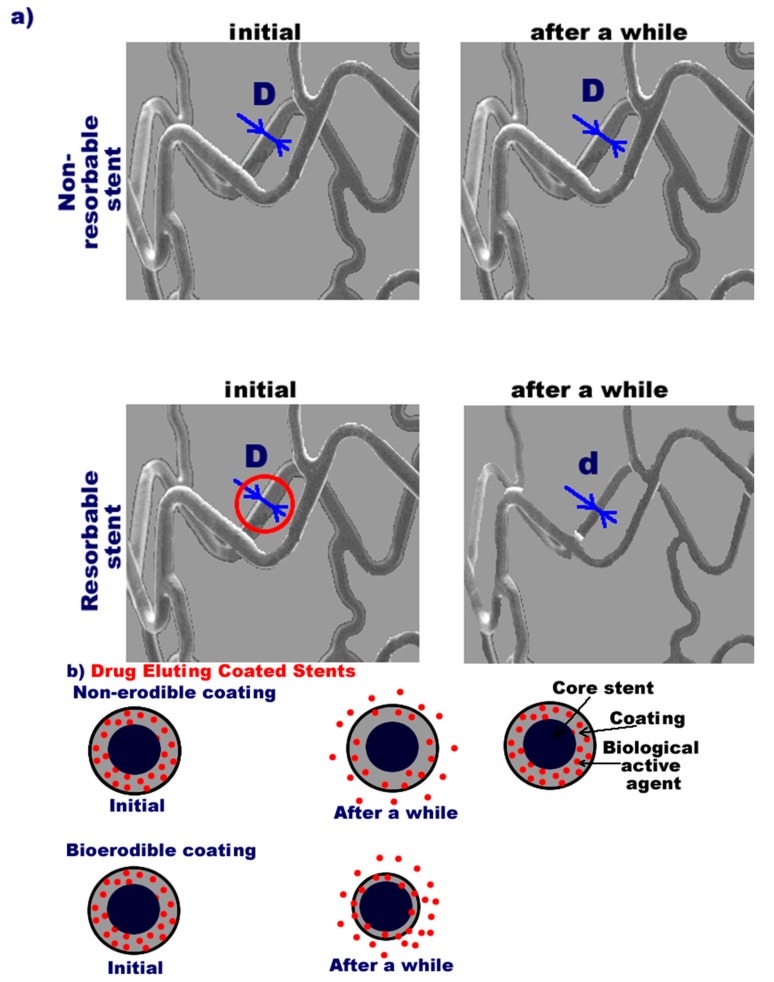
Schematic behavior of bioresorbable and non-bioresorbable stent after implantation (**a**) and mechanisms of drug elution (**b**).

Figure 2a schematically presents the behavior of non-resorbable and resorbable stent after implantation. Ideally, the non-resorbable stent should remain unchanged, no resorption or deposition should appear in time while, the resorbable stents degrade fully with a proper rate. It is important to mention, that there are classic resorbable stents (the stent is homogeneous) and coated stents with only resorbable coating. When the coating is loaded with a biologically active agent, it is released during degradation. Depending on the coating material, the release of drug can have non-bioerodible or bioerodible character according to the Figure 2b. In case of non-bioerodible mechanism, the drug release occurs due to diffusion processes otherwise it correlates with the degradation of the stent coating.

The manufacturing of these implants is based on the application of biocompatible biodegradable metals and alloys (Fe, Mg, Zn) and polymers (poly-L-lactic acid, poly-L-glycolic acid, polyorthoester, polycaprolactone, fibrin, hyaluronic acid, phosphorylcholine, or polyethylene oxide/polybutylene terephthalate (PEO/PBTP) [7,23,54]) able to be resorbed in the human body after several months of implantation. This implant property provides the greatest advantage of biodegradable stents as it allows for avoiding the permanent stay of the foreign material inside the human body helping to overcome the foreign body reactions, avoid complications and save expenses related to the re- or explantation of stents. Particularly important groups of patients benefitting from the new technology are old people, children, or diabetics having the most serious problems due to the repeated surgeries. In an ideal case, bioresorbable stents implanted into the human vessel undertake the lacking mechanical support for the healing period of time and degrade completely during 12 to 24 months. The properties and the degradation behavior of bioresorbable stents have to be predictable within a defined time interval. A stent material itself and its degradation products must be biocompatible and non-toxic ensuring the highest safety level for patients.

Patent US9878073 [105] demonstrates an example of such bioresorbable stents eluting nitric oxide (NO). The stent is comprised of three main key design elements: a bioresorbable scaffold, a bioresorbable polymeric coating layer(s), and NO-releasing nanoparticles incorporated in the bioresorbable polymeric coating layer and optionally also in the scaffold. The NO-releasing nanoparticles are made of non-toxic biocompatible and biodegradable materials. For example, a chitosan polymer and optionally a sugar [106,107].

### 3.1. Metallic Bioresorbable Stents

Magnesium (Mg), iron (Fe), and zinc (Zn) containing alloys were the first metals used for fabrication of biodegradable stents having sufficient mechanical characteristics and appropriate biocompatibility [27,28].

#### 3.1.1. Mg Stents

Despite multiple advantages of Mg as bioresorbable stent material (excellent biocompatibility and bioresorbability in the human body), the major hurdle disturbing its application consists in too fast and inhomogeneous degradation in the physiological milieu. Mg alloys have low corrosion resistance in aqueous environments containing halide ions. Mg implants in such medium undergo failure because of excessive hydrogen production, which forms gas patchy cavities and pH increase in neighborhood of the implant. The corrosion tendency of the Mg-containing alloys is correlated with its phase composition. In the solution containing Cl-, the attack of large numbers of active anion ions interacting with the implant surface leads to the pitting corrosion [108,109].

The actual research is focusing on the creation of a controlled degradation profile of Mg stents using different alloying elements, development of surface coatings or surface treatments, and adjustment of the stent geometry. Several patents (US 2017/0157300 A1 [110], US8915953B2 [111], WO2013024125A1 [112]) related to the first two methods could be found. However, the proposed inventions did still not lead to the commercialization of new Mg stents. Several works (US 2006/0271168 [113], 2009-0240323 A1 [114], US 2010-0076544 A1 [115], and US 2011/0076319 [116]) propose the concept of special coatings inhibiting the degradation rate of Mg alloys as a basic stent material. As biodegradable polymer coatings poly(lactide), polyhydroxyalcanoate (especially polyhydroxyvalerate), polycaprolactone, aliphatic polyester, aromatic copolyester, and polyesteramide can be used. In EP 2415489 B1 [117], a stent coating containing vaccination crystals and lipophilic substances as additives is presented. The proposed measures should reduce the coating permeability for corrosive medium. The functional principle of this technology is still not validated and no products utilizing its application are known.

Another problem of Mg application in stent manufacturing is related to the release of hydrogen as result of the degradation process leading to inflammation and systemic toxicity [118]. Ma et al. [119] studied magnesium-based alloys as potential, biodegradable cardiovascular stents and highlighted that magnesium release had a significant impact on vascular smooth muscle cells (SMCs). Based on their work, low concentrations of Mg^2+^ (<10 mM) increase the cell viability, proliferation rate, cell adhesion, cell spreading, cell migration rate, and actin expression, but at high concentration (40–60 mM) adverse effects can be observed (coagulation and inflammation but also SMC proliferation is inhibited). Kirkland et al. [120] investigated the degradation behavior of about 30 different Mg alloys in contact with simulated body fluids. This study has shown that the alloying of Mg with Zn, Ca, and rare earth elements like cerium (Ce), lanthanum (La), or neodymium (Ne) significantly influence the degradation rate and behavior of Mg. The alloying elements Zn, zirconium (Zr) and rare earth elements can lead to the improvement of strength and forming behavior as well as inhibit the degradation of Mg in simulated body fluid - SBF [28,121,122]. By such a way, it is possible to adjust the required properties of Mg depending on application and to find a compromise between the necessary mechanical properties, degradation rate, and biocompatibility. Another promising way for controlling the degradation rate of Mg stents are coatings. Despite of the rapid development of stent technologies, no optimal solution for the adjustment of Mg degradation exists. The results of different studies related to the properties of Mg stents are summarized in the Table 1.

The first Mg stent available on the market is balloon-expandable AMS-1 BDS (AMS-1, Biotronik AG, Bülach, Switzerland) composed from Mg (up to 93%) and rare earth metals (up to 7%) [127]. The stent has a strength-to-weight ratio comparable with that of stainless steel L316 and strong aluminum alloys. Pre-clinical assessment indicates the rapid endothelialization of AMS-1 and too high a rate of degradation (about 60 days) into inorganic salt. The AMS-2 stent uses a different magnesium alloy, resulting in the stent having a higher collapse pressure as well as a slower degradation time. The AMS-3 stent (DREAMS–Drug Eluting AMS) is a modification of the AMS-2 stent and is designed with the aim of reducing neointimal hyperplasia by incorporating a bioresorbable coating based on poly-L-lactic acid (PLLA) for controlled release of an anti-proliferative drug, and diminishes the rate of body-stent degradation at the initial stage. The first-in-man BIOSOLVE-I trial assessed the safety and performance of this first-generation drug-eluting magnesium-based BRS in 46 patients with 47 lesions at five European centers [127].

Corrosion properties of matrix composed from Mg and ZnO powder by a spark plasma sintering technics were investigated by Cao et al. [128]. The results indicate that Mg-10 wt% ZnO composite exhibits a lower corrosion rate compared to pure Mg and is promising as temporary implant. Lewis [129] presents a critical review of the methods to reduce the bio-corrosion rate of Mg and Mg-based alloys, and to define the way of decreasing the in vivo resorption time, i.e., improving the clinical efficacy of Mg-based grafts. The resorbability of magnesium alloys can also be reduced by salinization. Patil et al. [130] showed the ability of alkylsilane self-assembled multilayer coatings to reduce several fold the rate of Mg corrosion, demonstrating their good cytocompatibility and great potential of these coatings on developing bioresorbable Mg devices, including stents. In the invention WO2019043394, a bioresorbable metal alloy particularly suitable for the formation of bioresorbable medical devices, for example stents, is described. The metal alloy essentially comprises 3.2% to 4.8% by weight lithium, 0.5% to 2.0% by weight yttrium; and the balance being magnesium, in addition to any trace elements. The metal alloy can be drawn into a wire, which can be shaped into a stent scaffold. The stent can be produced using one or more stent scaffolds together with one or more bioresorbable polymer connectors, for example formed from poly(lactide-co-glycolide) (PLGA) [131]. There are several reviews on biodegradable metal stents [54,132] in which the main data concerning their properties are collected.

#### 3.1.2. Zn Stents

Zinc is proposed as an exciting new biomaterial for use in bioresorbable cardiovascular stents. It shows sufficient mechanical and biological characteristics required for optimal stent performance. Zinc and its alloys show the appropriate rate of degradation for stent application (0.02 mm/year [132,133]). The review [132] compares bioresorbable materials and summarizes progress towards bioresorbable stents. It emphasizes the current understanding of physiological and biological benefits of zinc and its biocompatibility. Finally, the review provides an outlook on challenges in designing Zn-based stents of optimal mechanical properties and biodegradation rate. Very prospective results were presented by Bowen et al. [133]. They confirmed the suitability of zinc and its alloys to be used as bioresorbable stent material having a good biocompatibility and mechanical characteristics while degrading slowly at an ideal rate of tens of micrometers per year. In principle, Zn and its alloys can help to avoid many problems associated with Mg and Fe because pure Mg has a corrosion rate 10 times faster than Zn ((300–600) µm per year [134]) while the corrosion of iron leads to non-bioresorbable iron oxides.

In human physiology, zinc is a crucial oligoelement playing important catalytic, structural, and regulatory roles within the cells [135]. Zn has a good tolerance to most of the tissues but relatively little is still known about its corrosion, toxicity, and biocompatibility in connection with its application as a stent material. Nowadays, these issues are under consideration [132]. The role of the main product of zinc biodegradation (Zn^2+^) in numerous fundamental cellular processes is considered in [136]. The formation of ZnO is beneficial because it induces a slightly antimicrobial activity, which can avoid biofilm formation [59,137,138,139,140].

The data of comparative testing the inflammatory reaction on pure Zn and different Zn-Al alloys demonstrated that viability of cells at the interface decreases from high-grade Zn (~99.7%) to Zn-Al alloys with the increase of aluminum concentration [141]. Clinically relevant long-term in vivo studies with the aim of characterizing late stage zinc implant bio-corrosion behavior were conducted by Drelich et al. [142]. Zinc oxide, zinc carbonate, and zinc phosphate are the main components of corrosion products surrounding the Zn implant. The obtained results support the predictions that zinc could be a suitable material for manufacturing biodegradable stents. The degradation rate of stents is strongly dependent on the surface structure, properties, and the presence of defects (bio-corrosion rate increases with the increase of their density). The degradation of Zn depends on the quality and stability of the oxide film formed on the stent surface [143].

Analysis of mechanical properties of Zn and Zn-based alloys regarding the general requirements for stent application [132,144] shows that when using conventional alloy families and processing methods, it is possible to produce a Zn-based material. This includes Zn-Mg, Zn-Ca, Zn-Sr, Zn-Al-Cu alloys and pure Zn with appropriate mechanical characteristics, in particular, ultimate tensile strength (UTS) in the range of 20–440 MPa, and elongation to failure 10–65% for stent application. The influence of severe plastic deformation on the structure and mechanical characteristics of the zinc alloy with 1 wt% of Mg is presented by Jarzebska et al. [145]. These results indicate that the plastic deformation leads to the grain refinement, and mechanical properties satisfy the requirements for bioresorbable stent applications. High Zn compatibility to magnetic resonance imaging is an additional advantage of this promising candidate for biodegradable stents as compared to iron- and magnesium-based stent materials. The efforts in the development of new Zn-based stent materials are only at the beginning and an increasing number of scientific groups is working on this topic [133,142,145,146,147,148,149,150,151]. The results of the different studies related to the properties of Zn stents are summarized in Table 2.

#### 3.1.3. Fe Stents

Pure iron and iron-based alloys play a special role in the research on bioresorbable stent technology [30,31]. Iron stents possess superior radial strength (due to its higher elastic modulus) [7], satisfactory mechanical characteristics [7], are non-toxic and may inhibit neointimal hyperplasia [27]. The main problems related to the application of iron as a stent material consists in the slow degradation kinetics (0.1–0.2 mm y^−1^) and its oxides as corrosion products, which are not metabolized at an appreciable rate, therefore, reducing the cross section of lumen and altering the integrity of the arterial wall [30].

The invention US2016263287 discloses an absorbable iron-based alloy stent covered by biodegradable polyester (average molecular weight of between 20,000 and 1,000,000 and a polydispersity index of between 1.2 and 30). The proposed combination of materials ensures a controllable degradation rate within a predetermined period of time. Following implantation into the human body, the degradable stent serves as a mechanical support at an early stage. It then gradually degrades, being metabolized and absorbed. During the process of degradation, minimal or no solid product is produced. Ultimately, the configuration of the lumen with an implanted stent as well as the systolic and diastolic functions thereof returns to their natural states.

### 3.2. Polymeric Bioresorbable Stents

Biodegradable polymeric materials show great promise in different medical applications allowing local delivery of biologically active agents and drugs. Shukla et al. [154] emphasizes the state-of-the-art of biodegradable polymers and polymeric nanostructures, and discuss their future perspectives. Poly-L-lactic acid (PLLA) is the best-known biodegradable polymer, often used in manufacturing biodegradable stents (BDS). PLLA proved to be highly biocompatible [155]. PLLA is metabolized via the Krebs cycle over a period of approximately 12 to 18 months into carbon dioxide and water without toxic degradation products. PLLA-based biodegradable coronary stents have initially been reported by Igaki-Tamai et al. [26] as the first fully degradable stent in the world (Kyoto Medical Planning, Kyoto, Japan). This event was followed by further development of bioresorbable polymeric stents with regard to their design and drug delivery function. Intensive research on the improvement of functional characteristics and biocompatibility of PLLA as well as poly(lactide-co-glycolide) (PLGA) has been conducted over the last years. Other PLLA-based stents, for example Elixir (Sunnyvale, CA, USA), ARTDIVA from Arterial Remodeling Technologies (Noisy le Roi, France), Tissue Gen (Dallas, TX, USA), and others [23,154], are also under investigation. The extensive discussion of the related results can be found in several reviews [21,22]. Despite the multiple advantages, polymeric biodegradable stents still demonstrate pure mechanical properties disturbing their application in patients. The clinical studies of everolimus eluting PLLA stent Absorb-BVS-System (Bioresorbable vascular scaffold; Chicago, IL, USA) have shown its safety with a good mechanical support during the first 3 months of implantation. After this time, mechanical strength diminished rapidly. Pharmacological properties and resorption dynamics of the stent were studied by Shukla et al. [154], who also highlighted the opportunities and challenges in the field.

Except for PLLA and PLGA polyhydroxycarboxylic acids, poly(3-hydroxybutyrate) [156] and poly(ε-caprolactone) have been used as well-known biodegradable polymeric materials for manufacturing stents in the research. Phosphoryl choline (PC) and poly(vinylidene fluoride)-hexafluoropropylene (PVDF-HFP) belong to the class of biomimetic polymers and are further applicants for DES of second- or third-generation [157]. The REVA stent (REVA Medical, San Diego, CA, USA) is a poly (iodinated desaminotyrosyl-tyrosine ethyl ester) carbonate stent with sirolimus and a slower drug release pattern [23]. BDS (Bioabsorbable Therapeutics Inc., Menlo Park, CA, USA) presented a biodegradable stent out of poly-anhydride ester combined with salicylic acid and sirolimus. This combination provides both anti-proliferative and anti-inflammatory properties [23].

Several types of bioresorbable stents coated with durable polymers (*Taxus Liberté* coated by styrene-b-isobutylene-b-styrene, *Endeavor Resolute* with Biolinx polymer coating and *Xience V* stent covered by fluoropolymer) were tested. The results of comparison for safety and efficacy of stents with biodegradable versus durable polymer coatings are presented by Lam et al. [158].

The invention WO2019043384 [159] provides bioresorbable polymeric stents made from polymer blends containing polyhydroxyalkanoates (PHAs). The patent proposes two material compositions for stent manufacturing: a) 40 wt% PHA copolymer comprising two or more different medium chain length hydroxyalkanoate monomer units and b) 60–95% PHA homopolymer containing a short chain length hydroxyalkanoate monomer unit or a polylactide (PLA).

Various polymers with different properties and special resorption rates are available for medical purposes, many of them being suitable for stent manufacturing. The most important problems, such as poor mechanical support, inadequate degradation rate, as well as generation of harmful fragments [160], have to be overcome in order to enable successful clinical use and commercialization.

### 3.3. Comparison of Bioresorbable Metal and Polymer Stents

In spite of challenges faced when choosing stent materials, it seems that metals have several important advantages over polymers: polymers exhibit lower Young‘s modulus (0.2–7.0 GPa) than metals (54–200 GPa), and metal stents are considered to be better than polymer grafts in terms of mechanical performance [132] with comparable other characteristics. Polymers were compared with Fe- and Mg-based metal grafts in review [132]: (i) exhibit radial force similar to those of stainless steel [161] and cobalt chromium stents [162]; (ii) demonstrate the profile required for successful deliverability of scaffold [7]; and (iii) demonstrate required rate of degradation [127]. However, low ultimate tensile strength by polymers requires greater struts thickness than those of metals. This led to the inability of complete expansion with balloon dilatation. Considering that restenosis rates in polymer stents are similar to that of BMS, the latter has the advantage. Ho et al. [102] provides contemporary data on the evolution of coronary artery stents from BMS through drug-eluting stents to bioresorbable stents. Their manuscript highlights that BMS are suitable for the cardiovascular application and are strongly dependent on the structure platform, size, length, and strut thickness. The development of newer stents, with thinner struts and covered with bioresorbable polymers can present an important improvement, especially because of the reduction of the restenosis rate. From an evolutionary point of view, the first reduction of the restenosis rate was achieved by using thinner struts and new metal compounds, later by using drug-eluting stents and polymer coated stents [32,33,34,102].

## 4. Drug, Nanoparticle, and Gene-Eluting Stents

### 4.1. General Aspects

Drug-eluting stents are stents with drug-eluting functions, being realized by means of an anti-inflammatory/antithrombotic drug-containing polymer coating or direct immobilization of drugs on the stent surface. Since the first approved DES, CYPHER^TM^ in 2003, different stents have been developed to ensure quick endothelialization, low proliferation of Smooth Muscle Cells (SCMs) and to avoid late in-stent restenosis. Although, the first generation of DES loaded with sirolimus and paclitaxel have shown reduced in-stent restenosis rates, these stents are still associated with a risk of late stent thrombosis due to the hypersensitivity [163]. Biodegradable polymer coating is designed in order to avoid inflammation and delayed vascular healing as compared to the use of durable polymers.

In the second generation, the development of zotarolimus- and everolimus-eluting stents have further reduced that risk exhibiting lower hypersensitivity, high flexibility, acceptable recoil and better compliance [163].

The third generation of DES belongs to the bioresorbable drug-eluting vascular scaffolds (BVS), which disappear or degrade completely after a certain time in the vessel [164,165,166,167,168]. Just as metal DES, BVS have the advantage of no long-term limitations of permanent vessel caging and possible malapposition (incomplete stent apposition), significantly reducing risks of late restenosis, neoatherosclerosis, thrombosis, and local inflammation [169,170,171,172,173,174,175]. The whole polymer stent may be used as drug reservoir [21] and exhibits difficulties with implantation in accurate position within vessels [21,176]. The resorbable metal stent (Biotronik, Berlin, Germany), which is composed of magnesium and some other rare metals, is the first bioresorbable metal stent implanted in humans. The device showed high mechanical strength and properties similar to other metal stents. The stent resorption is completed within four months without causing any significant inflammatory response [171,175,177,178,179,180]. A PowerStent^®^ Absorb prototype (blended ACP with high molecular weight PLLA to address two major challenges in BDES development: inferior radial strength and biocompatibility) was manufactured and tested in vivo in the coronary artery of a porcine model, which reduced stenosis, recoiling and inflammation [181].

Furthermore, stents can be improved by using DNA, siRNA, and miRNA [182,183,184,185,186,187,188,189,190,191] as well as nanoparticles [164,192,193] instead of drugs. For example, Zhao et al. [164] developed a novel coating method using sirolimus-loaded PDLLA (Poly DL Lactide) nanoparticles applied on a 3D-printed PLLA biodegradable stent with the result of a better inhibition effect on smooth muscle cell proliferation than on endothelial cell proliferation.

Currently, there is a tendency to fabricate polymer-free drug-coated stents (PF-DES). Examples for this are the stainless steel sirolimus-containing stent VESTAsyn (MIV Therapeutics, Atlanta, GA, USA), the stainless steel BioFreedom stent (Biosensors) coated with Biolimus A9, and the polymer-free cobalt chromium Amazonia Pax stent (Minvasys, Genevilliers, France) with paclitaxel [102]. The first step is the modification of the stent surface aiming at creating the sites of drug localization, and then drug deposition. The sites of drug localization may be a drug reservoir—a system of nano-, micropores, nanoparticles in a matrix compound on a stent surface. Several stents are described: (i) Yukon SS stent (Translumina, Hechingen, Germany) with microporous surface coated with sirolimus; (ii) cobalt-chrome stent Cre8 (CID Vascular, Saluggia, Italy) with nanoparticles contain ultra-thin passive carbon coating; (iii) OPTIMA (CID, Saluggia, Italy) tacrolimus-coated stainless steel stent with the grooves on the abluminal surface as a drug reservoir; (iv) Amazonia Pax (Minvasys, Gennevillieres, France) cobalt-chromium stent with paclitaxel applied as microdrops on the abluminal surface. Due to the absence of polymer remnants released by the degradation process, polymer-free stents may be beneficial in decreasing the rate of stent thrombosis and inflammation reactions. Table 3 provides an overview of three generations of stents with common features. There are four materials used in BVS including PLLA, magnesium, polyanhydrides (salicylic acid and adipic acid), and polycarbonates (amino acids, e.g., tyrosine) where PLLA is the most investigated one [175,194]. In general, commonly used DES can be divided into (a) polymer-coated; (b) polymer-free; (c) gene-eluting [182,183,184,185,186,187,188,189,190]; (d) nanoparticle-eluting; and (e) bioresorbable.

### 4.2. Drug-Related Surface Modification

The achievement of optimal drug release kinetics and drug loading capacity are the most important challenges for DES. Burst drug release (elution of 90% of the drug amount within two days) in various PF-DES have been reported repeatedly. As a result, the desirable inhibition of neointima proliferation cannot be reached [198,200,201].

In order to overcome this barrier and obtain a sustained drug release kinetics profile of the DES platforms (elution of 60–70% of the drug amount within the first week and the remaining drug within 4–6 weeks), different physical and chemical methods of stent surface modification have been used (see Table 4). The coating techniques used for surface modification of stents include direct coating, crystalline coating, nanoporous or microporous surface coating, inorganic porous coating, reservoir-based coating, coating containing nanoparticles, and a coating of self-assembled monolayers. Direct coating is the technique to coat the drug onto the surface of the stent by immersing the stent into a drug solution followed by evaporation [202]. In crystalline coating, direct crystallization of the drug from a solvent on the stent surface leads to a partially crystalline or an amorphous drug-coated stent [203]. Nanoporous or microporous surface coating uses a sand blasting technique and mechanical modification [204]. Inorganic porous coating includes the coating of micro- or nanoporous biocompatible thin inorganic material on stent deposited by anodization technique [205]. Reservoir-based coating uses macropores (grooves, channels or holes) created by mechanical treatment on stents, which act as reservoir-based systems for drugs [206]. Nanoparticle containing coating on stent is a recent approach used for NPDES by coating nanoparticulate-based chemotherapeutics onto the stent platform [207,208]. Silica-based magnetic nanoparticles and carbon nanotubes are used as nanoparticulate systems [209]. Coating of self-assembled monolayers on stent surfaces are applied by a two-step deposition method: (a) immersion into solution and (b) dip evaporation [210]. The most frequently used method is surface modification through a creation of micropores by sandblasting or mechanical modification. The first microporous surface PF-DES platform used in clinical studies was the Yukon DES stent [200]. One example of a micro-patterned drug delivery stent is presented in the patent US8915957B2 [211]. It contains special arrays of micro- and nanostructures covering the stent surface in selected regions and providing dynamically controllable hydrophobicity for the whole stent. Additionally, some special options, namely a possibility to use a control devise able to vary the hydrophobicity of the structured regions, dynamical control of the stent shape in vivo as well as use of sensors for monitoring the fluid parameters have been presented in this work.

DES mostly use polymer coatings to incorporate pharmacologic agents. These are (i) non-biodegradable polymers, such as phosphorylcholine (PC, Endeavor^®^stent, Medtronic), C10, C19 and polyvinyl pyrrolidone (PVP, BioLinx polymer system), parylene C, polyethylene-co-vinyl acetate (PEVA), poly-n-butyl-methacrylate (PBMA) (CYPHER^TM^ stent, Cordis), poly(styrene-b-isobutylene-b-styrene) (TAXUS^®^ stent, Boston Scientific), PBMA and polyvinylidene fluoride hexafluoropropylene (PVDF-HFP) (Xience V^®^ stent, Abbott Vascular; PROMUSTM ElementTM, Boston Scientific). (ii) Biodegradable polymers, such as poly-lactide-co-caprolactone (PLC), and poly-lactide-co-glycolide (PLGA) (Supralimus 181and Infinnium 181 stent, Sahajanand Medical Technologies), poly-L-lactic acid (PLLA) (Excel stent, JW Medical System) [190]. Some polymer coatings, which are recently being used, are summarized in Table 5.

### 4.3. Drugs Used in DES

DES utilize different drugs with anti-inflammatory, anti-thrombogenic, immuno-suppressive, and anti-proliferative effect mechanisms [218]. Thereby, “limus family” drugs are particularly evaluated. Table 6 summarizes the commonly used drugs in DES according to their binding target, structural formula, and mode of action.

### 4.4. Drug Delivery Mechanisms

The controlled drug delivery mechanisms [219,220,221,222,223,224,225] can be classified as either physical or chemical mechanisms, or their combination. Physical mechanisms include diffusion of drug molecules through a polymer layer, dissolution, or degradation of polymer matrix controlling the drug release rate, use of osmotic pressure for drug release and use of ion exchange for ionized drugs. The chemical mechanisms, however, are based on breaking covalent bonds that connect drug molecules to a delivery vehicle, such as polymer chains, by either chemical or enzymatic degradation. Physical mechanisms have an advantage over chemical ones as they allow for controlling the drug release kinetics by the drug delivery system itself. Furthermore, there is no need to chemically modify the drug molecules, such as in chemical mechanisms.

Diffusion: in the reservoir devices, the drug reservoir is covered with a thin polymer layer, which serves as a rate-controlling membrane. In the matrix (or monolithic) devices, a drug is usually dispersed inside the polymer matrix. Its release into the environment occurs without any rate-controlling barrier layer. In CYPHER^TM^ (Cordis) [226] and Taxus^®^ (Boston Scientific) [227] stents diffusion-controlled mechanism is used.

Dissolution or degradation: dissolution-/degradation-controlled drug release is based on decomposition of a polymer membrane encapsulating the drug reservoir or a drug-containing polymer matrix itself. Examples of stents, in which the dissolution or degradation mechanism is used, are Achieve (Cook Inc) [228,229], ACS Multi-Link^TM^ stent (Guidant Corp.) [230], ConorMedstent^TM^ (ConorMedsystems) [231], and Janus CarboStent^TM^ (Sorin Biomedica) [207].

Ion exchange can be used very effectively for controlled release of ionized drugs, which bonds to the matrix through electrostatic interactions. Ion exchange mechanism is employed in the BiodivYsio stent (Biocompatibles International) [232].

The osmosis-based controlled release devices consist of an inner core containing drug and osmogens, coated with a semipermeable membrane. As the core absorbs water, it expands in volume, which pushes the drug solution out through the delivery ports. Osmotic pumps release the drug at a rate that is independent of the pH and hydrodynamics of the dissolution medium [219].

Prodrug approach is based on chemical (e.g., hydrolysis) or enzymatic degradation in the body. The drug release kinetics is likely to be affected by the parameters, such as pH and the enzyme concentration, which cannot be controlled by the system itself [219].

## 5. Mechanical Aspects of Stents

The mechanical characteristics of the material (elastic (Young’s) modulus (YM), yield strength (YS), ultimate tensile strength (UTS), and elongation) define the characteristics of the stent (radial strength, acute and chronic recoil, axial and radial flexibility, deliverability, profile, and lifetime integrity). The requirements to mechanical characteristics of the stent material are rather contradictory: a high value of YM is needed to reduce stent recoil; the combination of a high UTS (>300 MPa) and low YS (∼200 MPa) value is preferred for the design of stents; high UTS and high YM is needed to increase the stent’s radial strength. Low YS is required for ease of crimping the stent onto a balloon and then expanding them during deployment. It is obvious that the more these characteristics are achieved, the better the stent will be. Various, influential, sometimes conflicting factors affect one or more of these characteristics: materials, manufacturing methods, general shape of the stent/stent design and size, struts shape, size and number. Materials are responsible for corrosion resistance, biocompatibility, radio-opacity and—along with manufacturing methods (laser cut, water-jet cutting, photoetching)—the apparition of the artefacts. When materials selection is combined with stent design, strut shape, and size, mechanical properties are influenced, such as radial strength and recoil.

Open and closed cell design: general shape of the stent (coil, tubular mesh, slotted tube) and bridging between rings (peak-to-peak, peak-to-valley, and mid-strut-to-mid-strut connections) can influence flexibility, radial strength and scaffolding (ability to support tissue; thus, preventing prolapse) [233]. Slotted tube stents design can be either open or closed cells. Closed cells are only peak-to-peak connected; they provide optimal scaffolding, high radial strength, low plaque prolapse. When bending the stent, its surface is more uniform, which leads to a more uniform drug concentration. Contrary to closed cells, open cell stents have some or all internal inflection points of the structural members not connected by bridging elements. Thus, the unconnected structural elements contribute to the longitudinal flexibility. In turn, at bends, gaps are formed on one side, so the amount of drug is lowered (low drug delivery capability); on the opposite part, the pinch creates a high drug concentration. Hybrid design that includes both closed and open cells in different parts of the stent can address some of these issues. In synthesis, the selection of one of the two options affect scaffolding, drug delivery capability, conformability, and mechanical properties. Lower size of the stent (in length and/or diameter) has been reported to decrease the risk of restenosis. Xu et al. [234] studied the effects of vascular dynamic bending (VDB) and vascular pulsation (VP) in alternating stress states of an open-cell design (Endeavor™ stent). They found that stent fracture occurred more frequently as a result of VDB with the predicted fracture position located in the bridging struts of the stent, where the maximal stress of the stent about 590 MPa was recorded. In the axial direction, the stress is mainly distributed in the middle loops of the stent, corresponding to the maximal bending deformation.

The stent presented in the patent US6908480B2 [235] has at least two different patterns along its longitudinal length, such as a closed cell and an open cell design. The stent is made from nickel/titanium, titanium, stainless steel or a noble metal. The different patterns are joined by varying articulations including a W-pattern and S-pattern. The stent has at least two coatings over the base structure, the coating depth not exceeding ten microns.

Strut thickness: strut thickness has been decreased over time, as thinner struts have been associated with a lower late luminal loss and less neointimal volume obstruction after stenting, possibly a result of less stent-induced arterial injury and inflammation [236,237,238]. Reducing strut thickness results in increased conformability and deliverability, but less radio-visibility and affected mechanical properties. Materials such as cobalt-chromium alloys can be used to deal with these issues. Round cross-section struts are preferred due to their smoothness to square cross-section struts. Smaller struts are less prone to fracture than bigger struts. Fewer struts induce a lesser chance of restenosis compared to more struts.

Depot stents design: depot stents design optimization has been performed by Hsiao et al. [239]. Unlike drug-eluting stents, the depot stent does not need to be surface-coated. They observed that creating reservoirs on the stent struts lowers the mechanical properties. Computational modelling of the depot stent using finite element analysis (FEA) leads to the best compromise in order to increase the drug capacity without significantly comprising its mechanical integrity. The depot stent was an L-605 cobalt–chromium balloon-expandable stent, on whose strut micro-sized drug reservoirs were created in order to investigate their effects on the stent mechanical integrity.

The FEA simulation was then conducted to investigate the effects of the reservoir location on the mechanical integrity of the depot stent. Equally spaced cylindrical reservoirs were created on the depot stent. The stress analysis identified the most fracture-prone locations of the stent as being the strut crown. By simply not performing holes (drug reservoirs) in the crown, the maximum equivalent plastic strain was reduced by 9% and the strain distribution was spread out even more uniformly than the case. Schiavone et al. [233] compared the mechanical performance of metal (Xience) and bioresorbable polymer (Elixir) stents during the process of crimping and deployment. High levels of stresses were observed in both stents following their deployment in the artery showing maximum von Mises stresses in the U-bend areas with a value of 935 MPa for Xience and 95 MPa for Elixir stent. Zhao et al. [240] also obtained a higher concentration of plastic strain on the curved crowns of the stent. Plastic strain concentrations occurring at the crown junction of the stent may be the cause of the stent fracture. Mehta et al. [241] highlighted that deformation mechanisms of Nitinol are more complex than the conventional modes of plastic deformation in traditional alloys. Therefore, future development of finite element models must incorporate effects of transformational strain, phase redistribution, and plastic strain to provide higher fidelity predictions of Nitinol stent performance in vivo.

The patent US9532888B2 [238] presents the usage of above mentioned depot design for incorporation of radiopaque markers into depots available on the stent surface.

Overlapping stent design: Xu et al. [242] studied the interaction types and location of overlapping stents. It was found that all the overlapping contact patterns between struts are edge-to-edge or edge-to-surface with no surface-to-surface contact pattern. This phenomenon is mainly caused by the non-uniform deformation of the stents in the radial direction during the implantation and their tubular structure. After expansion of the second stent, the contact pressure is primarily concentrated on its edges, so that the failure of an overlapping stent frequently occurs along the edges. Mehdizadeh et al. [243] created a nitinol overlapping open ring with asymmetrical, intermeshed saw-tooth design—called recoil-resilient ring (RRR)—to be utilized standalone or potentially integrated with existing stents for reducing the mechanical failure due to recoil. These teeth can slide on top of each other during expansion but interlock afterwards when pressure is released. FEA compression tests indicate 13 times less reduction of the cross-sectional area of the RRR compared with a typical stainless-steel stent and perfect elastic recovery of the RRR after removal of the pressure as compared to the remaining plastic deformations of the stainless-steel stent.

**Figure 3:**
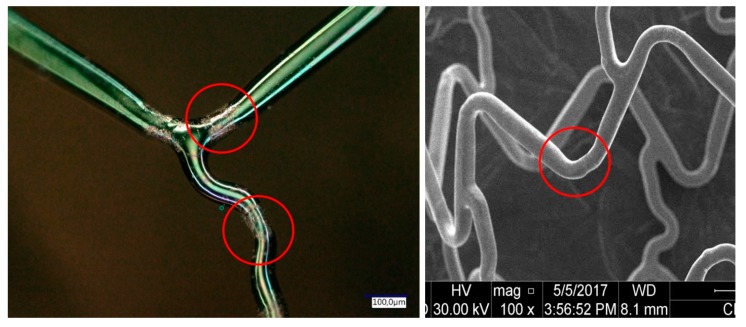
Thinned area in the strut formed after stent expansion.

Strut crown: possible weakness points (that are due to the plastic deformation) may appear in the strut crown; these weakness points can promote strut’s fracture (Figure 3).

Strut crowns and struts curved areas are indispensable as they allow the stent deformation by deployment. The smaller the curvature radius of the crown, the higher the deformation and, consequently, the risk of coating flaws formation. In order to minimize the impairment of a coating integrity, the following measures can be evaluated using numerical simulation:

− reducing the number of crowns;
− curving of the linear parts of the struts with maximally high curvature radiuses permitting further stent deformation and deployment;
− design improvement of the crowns.

**Figure 4:**
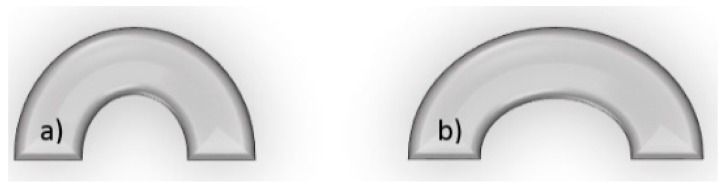
Crown design: (**a**) low and (**b**) high radius of curvature in the middle area of the crown.

The last effect can be achieved, for example, using larger curvature radiuses (Figure 4a) or by introducing a flat area or an area with a higher curvature radius (Figure 4b) in the middle of the crown. The assumed reduction of the distributed strain should occur due to the known inverse proportional relationship between a strain and curvature radius.

**Figure 5:**
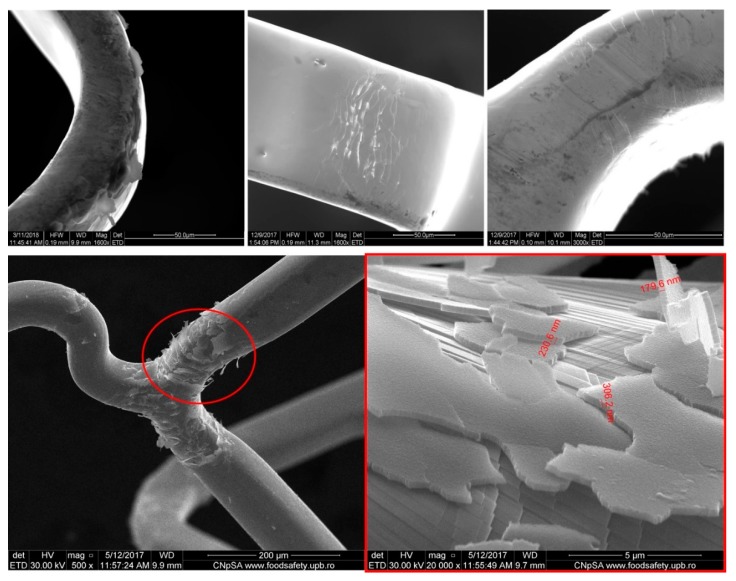
Inflated titanium oxynitride coated stent with coating flaws visible on all crowns (cracks, delamination, and possible peeling off in selected areas).

Coating integrity plays a major role for reliability and safety of the stent device. Effects, such as cracking, delamination, and peeling off a stent surface are associated with serious health risks explained by a distribution of small coating parts by the blood flow. This problem illuminates a high need to tailor a stent design in order to reduce the probability of mechanical damage of the coating and the whole stent itself. Due to the high mechanical stresses, it is important to consider the areas of the largest plastic deformation, i.e., the strut crowns. Figure 5 depicts the formation of coating flaws predominantly in the areas of stent crowns supported by cracks and visible delamination.

Coated stents were investigated by Shi et al. [244] revealing that the coating retained its original integrity after being crimped to ~Φ1.4 mm followed by expansion to ~Φ3.1 mm with no obvious delamination or peeling-off detected. However, at the locations of larger plastic deformation—on crown junctions—nano- and micro-sized cracks were identified. Such defects on the stent surface can lead to localized corrosion, stress corrosion cracking, and stent fracture as a result of high residual stress concentration at strongly deformed locations.

Most stents available on the market have struts oriented along the longitudinal axis. Some of them, such as Taxus Liberté, have those oriented at an angle relative to the stent longitudinal axis (multi-angled struts) [236]. In future studies, it should be examined if using angled struts and some of the above described measures can help to achieve more homogeneous distribution of mechanical loadings, resulting in the better reliability of stents as medical devices.

## 6. Conclusions

Due to a very high mortality rate caused by cardiovascular diseases worldwide, and a promising approach of stent technology, researchers and clinicians are paying great attention to develop new materials, methods, and solutions in order to improve the clinical outcome of currently existing stent types, aiming at more safety for patients, and a higher success rate of cardiovascular treatments. In the frame of this review paper, different technologies of stent fabrication, especially related to coated, bioresorbable, as well as drug-eluting stents, have been considered. Since the appearance of the first stent, newer stent classes have been designed, including covered stents or bioresorbable stents demonstrating desired release of biological active agents able to control adhesion, cell differentiation, and tissue development, and having suitable physical–chemical properties and degradation rate. Despite the huge progress in the stent technology, no ideal stent exists until now. It is expected that some of the existing problems will be overcome in the close future, as we can especially remark in the numerous patents filled in the last few years.

## Figures and Tables

**Table 1:** Mg alloys used for stent manufacturing.

Mg–Alloy	Key Features	Ref.
Mg–Zn (up to 3% Zn)	Higher affinity of adsorption to the surface of Mg–Zn alloy with the increase of Zn concentration (up to 3%).	[123]
Mg–Y (1% Y)	Adsorption of peptides is slightly weakened compared to that on the clean Mg (0001) surfaces.	
Mg–Nd (1% Nd)		
Mg (3.5 or 6.5%)-Li (0.5, 2 or 4%)-Zn	Good mechanical properties, degradation behavior, cytocompatibility, and hemocompatibility. Enhanced mechanical properties—yield strength, ultimate strength and elongation (twice as compared to pure Zn) and corrosion resistance without losing the viability of the Human Umbilical Vein Endothelial Cells (HUVECS) and Human Aorta Vascular Smooth Muscle Cells (VSMCS).	[124]
Mg–Al alloy AZ61	Highly susceptible to stress corrosion cracking (SCC) as compared to Zn, which is highly ductile with limited susceptibility to SCC.	[125]
MgZnYNd (coated with arginine (Arg)-based poly (ester urea urethane) (Arg–PEUU))	Super corrosion retardation, high hemocompatibility, high cytocompatibility.	[126]
Mg stent (coated with phytic acid (PA)); heparin loaded PA and bivalirudin loaded PA	Effective control on corrosion rate, biofunctional effect, good hemocompatibility, inhibits platelets adhesion, promotes endothelial cells growth superior stents compared with the bare Mg stents, super-hydrophilic surface (the contact angle being very close to zero). Hydrogen evolution vs. immersion time exhibit a slightly linear release between 5 and 10 days as compared to uncoated samples where an exponential hydrogen release was noticed within this interval.	[124]

**Table 2:** Zn alloys used for stent manufacturing.

Zn-Alloy	Key Features	Ref.
Pure Zn	Stents maintained mechanical integrity while no severe inflammation, platelet aggregation, thrombosis formation, or intimal hyperplasia were observed in abdominal aorta of rabbits. Good mechanical integrity for 6 months. After 12 months of implantation, the degraded volume of the stents was 41.75 ± 29.72%.	[150]
Zinc wires coated with PLLA/MPS	Corrodes at half the rate of uncoated Zn. Reduction of the biocompatibility and increasing cell toxicity and neointimal hyperplasia takes place.	[151]
Zn-1% Mg and Zn-1% Mg-0.5% Ca	These zinc alloys can be considered as good candidates for biodegradable implants.	[152]
Zn-Li alloy	Increase of ultimate tensile strength from <120 MPa (pure Zn) to >560 MPa. In vitro corrosion was evaluated by immersion tests in simulated body fluid and reveal higher resistance to corrosion compared to pure Zn. Samples containing 4% Li have shown the best results.	[149]
Zn-3Cu-xFe (x = 0, 0.5 and 1 wt %) alloys	The mechanical characteristics and in vitro behavior of Zn-3Cu-xFe alloys are more suitable than that of Zn-3Cu alloys as candidates for biodegradable materials.	[153]
Zn–Al alloys (containing up to 5.5 wt% Al)	Important mechanical characteristics: Yield strength 190–240 MPa; ultimate tensile strength 220–300 MPa, elongation 15–30%, elastic ranges 0.19–0.27%. Intergranular corrosion of Zn–Al alloys and cracking related with corrosion are observed. Absences of necrosis traces, though chronic and acute inflammatory indications were present.	[147]

**Table 3:** Overview of drug-eluting stents.

Stent (Manufacturer)	Type/Generation	Drug	Material (Bulk/Polymer)	FDA	Trials	**Drug-Eluting Time**	**Ref.**
CYPHER**^TM^** (Cordis)	SES/First	Sirolimus	Stainless steel/Parylene C	2003	FIM (First-In Man), RAVEL, SIRIUS, E-SIRIUS, C-SIRIUS	80% of sirolimus elutes over ~30 days; remainder released by end of 90 days	[163,169,195]
Taxus^®^ (Boston Scientific)	PES/First	Paclitaxel	Stainless steel or platinum-chromium/Translute^TM^ polymer	2004	TAXUS I-VI, TAXUS ATLAS, PERSEUS	elutes over ~90 days	[163,169,195,196]
Endeavor^®^ (Medtronic Inc., Minneapolis, MN)	ZES/Second	Zotarolimus	cobalt-chromium/phosphorylcholine	2008	ENDEAVOR I–IV	80% during first 10 days	[195,197]
Xience**^TM^** V (Abbott Laboratories)	EES/Second	Everolimus	L-605 Co-Cr/Poly (vinylidenefluoride-co-hexafluoropropylene) (PVDF-HFP)	2008	SPIRIT I-IV (Standard Protocol Items: Recommendations for Interventional Trials I-IV)	80% during first 30 days	[195,197]
Axxion (Biosensors International)	PF-DES	Paclitaxel	316L SS	-	-	40–50% in the first week 100% after 4 weeks	[198]
Achieve (Cook Inc.)	PF-DES	Paclitaxel	316L SS	-	8 months DELIVER (DELiverability of the Resolute Integrity Stent in All-Comer Vessels and Cross-OvER Stenting) Clinical Trial	28% within 4 days 69% within 2 weeks	[198,199]
Amazonia PAX (MINVASYS)	PF-DES	Paclitaxel	L605 Co-Cr	-	Pax A and Pax B Clinical Study Design	60% within 48 h, 100% within 7 weeks	[198,199]
Biofreedom (Biosensors International)	PF-DES	Biolimus A9	316L SS	-	BioFreedom FIM	98% of drug within 4 weeks	[195,198]
Polymer-free DFS (Medtronic)	PF-DES	Sirolimus	Co-Cr, Tantalum	-	Medtronic RevElution Trial	N/A	[8,198]
Cre8 (Alvimedica)	PF-DES	Amphilimus	L605 Co-Cr	-	Clinical performance of CRE8 drug-eluting stent in all comer population (PARTICIPATE) (phase 4)	50% of drug on 1st day 100% within 3 weeks	[8,198]
JANUS (Sorin Biomedica)	PF-DES	Tacrolimus	316L SS	-	JUPITER I, JUPITER II	50% over 4 weeks	[195,198]
NANO + (LEPU Medical)	PF-DES	Sirolimus	316L SS	-	Clinical performance of nano plus sirolimus-eluting stents in patients with coronary artery disease	85% in 4 weeks	[8,198,199]
Supra-G (Cook Inc.)	PF-DES	Paclitaxel	316L SS		6 months ASPECT (Asian Paclitaxel-Eluting Stent Clinical Trial)	N/A	[198,199]
VEST Async (MIV Therapeutics)	PF-DES	Sirolimus	316L SS	-	9 months Vest Saync II Clinical Trial	100% in 3–4 weeks	[198,199]
V-Flex Plus (Cook Inc)	PF-DES	Paclitaxel	316L SS	-	6 months Clinical Trial	28% within 4 days 69% within 2 weeks	[198,199]
YUKON (Translumina GmbH)	PF-DES	Sirolimus, Probucol	316L SS		ISAR-TEST, ISAR-TEST 3, ISAR-TEST 4, ISAR-PEACE (Posthumous Evaluation of Advanced Cancer Environment	66% in 2 weeks 100% over 3 weeks	[195,198]
YINYI (Liaoning Biomedical Materials)	PF-DES	Paclitaxel	316L SS		Safety and Efficacy Registry of Yinyi Stent (SERY-II) (SERY-II)	42% in 24 h 100% in 9 weeks	[8,198]

SES - Sirolimus eluting stent, PES - Paclitaxel eluting stent, ZES - Zotarolimus eluting stent, EES – Everolimus eluting stent, 316L SS – 316L Stainless steel, L605 Co-Cr – L605 Cobalt-chromium, PF – Polymer free.

**Table 4:** Comparison of various surface modification techniques used in drug eluting stents (DES).

Coating	Technology	Advantages	Disadvantages	Ref.
Direct coating	Stent dipping into the drug solution followed by solvent evaporation	Ideal for drugs with a very low aqueous solubility	Limited loaded drug amount; burst drug release kinetics	[8,198,199]
Crystallization	Direct temperature-dependent or micro drop spray drug crystallization on the stent surface	Slower release than amorphous drug layers due to lower dissolution rate	Limited loaded drug amount; burst drug release kinetics	[8,198,199]
Nano-/micro-porous coating	Micro/nanopores on the stent surface produced by sandblasting or mechanical modification	Higher amount of drug loading; sustained drug release due to a longer diffusion time; rough surface induces early endothelialization	Possible release of aluminum oxide particles	[8,198,199]
Inorganic porous coating	Pores are localized in an inorganic coating on the metal stent surface	Reduction of platelet activation due to a decreased release of metal ions	Release of inorganic particles after implantation pose a major challenge	[8,198,199]
Macroporous drug reservoir	Drug reservoir in form of abluminal stent grooves, holes or channels	Single and multidrug loading; slower drug elution by barriers like nanopores	Release of ions may cause local irritation	[8,198,199]
Nano-particle coating	Surface coating with a porous composite matrix based magnetic silicon and carbon nanoparticles or self-assembled monolayers	High drug adsorption and flexibility of the nanoparticle coating; rapid endothelialization	Nanocarrier properties are critical since the polymer may trigger mild immune response	[8,198,199]
Drug filling/ internal coating	A drug coats an internal lumen of the stent, diffusing through abluminal microholes directly into the vessel wall	Slower drug elution by barriers like microholes	N/A	[8,198,199]
Self-assembled monolayers	Deposition of self-assembled hydrocarbon chains on a stent surface	Controlled release and rapid endothelialization	Low drug loading	[8]

**Table 5:** Common polymers used in DES.

Polymer DES Coating	Features	Ref.
Polylactic acid	Effective in short and mid-term follow-ups	[190,212,213,214]
Poly-l-lactic acid (PLLA)	Feasible, safe, and effective implantation	[190]
Poly (lactic-co-glycolic acid)	Slow releasing capability for hydrophobic drugs	[190]
Hyaluronic acid	Good degradation efficiency, enhances the proliferation and migration of endothelial cells	[190,215,216,217]
Polyzene-F	Highly biocompatible, anti-inflammatory, bacteria-resistant and pro-healing	[190]

**Table 6:** Drugs commonly used in DES.

Drug	Binding Target	Structural Formula	Mode of Action
Sirolimus	FK-506 Binding Protein 12	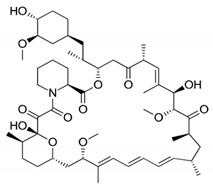	Anti-proliferative, immunosuppressive
Umirolimus/ Biolimus A9/ Biolimus/BA9	FK-506 Binding Protein 12	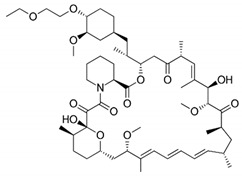	Immunosuppressive
Zotarolimus	FK-506 Binding Protein 12	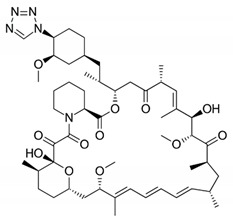	Anti-proliferative, immunosuppressive
Everolimus	FK-506 Binding Protein 12	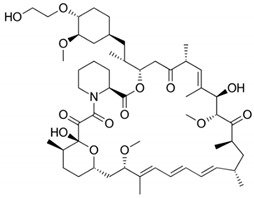	Immunosuppressive
Novolimus	FK-506 Binding Protein 12	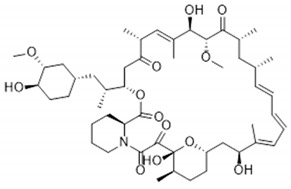	Anti-proliferative, anti-inflammatory
Tacrolimus	FK-506 Binding Protein 12	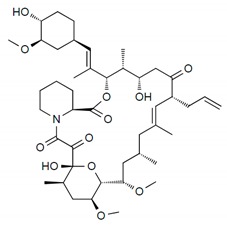	Anti-proliferative, immunosuppressive
Pimecrolimus	Macrophilin-12	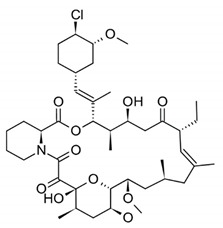	Immuno-modulating agent of the calcineurin inhibitor
Paclitaxel	Microtubules	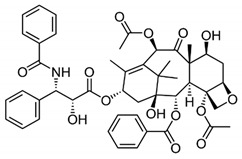	Anti-proliferative agent
Dexamethasone	Specific steroid-binding protein receptor	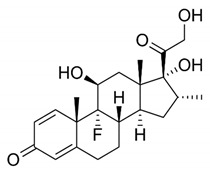	Anti-inflammatory
Curcumin	Microtubules	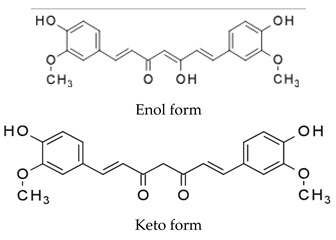	N/A
Terumo statin	--	-	Anti-proliferative
